# Enhancing wheat quality through color sorting: a novel approach for classifying kernels based on vitreousness

**DOI:** 10.3389/fpls.2025.1534621

**Published:** 2025-04-17

**Authors:** Jin-Kyung Cha, Hyeonjin Park, Youngho Kwon, So-Myeong Lee, Jeonghyun Kim, Woo-Jae Kim, Kwangho Park, Woosik Jang, Youngeun Lee, Byung Jun Jin, Kidong Han, Ki-Won Oh, Jong-Hee Lee

**Affiliations:** ^1^ Department of Upland Crop Sciences, National Institute of Crop and Food Science, Rural Development Administration, Miryang, Republic of Korea; ^2^ Technology Service Division, National Institute of Crop and Food Science, Rural Development Administration, Jeonju, Republic of Korea; ^3^ Department of Electronic Optical Development, DAEWON Global System Integration Co., Ltd., Chilgok, Republic of Korea

**Keywords:** wheat, color sorting, protein content, kernel vitreousness, quality

## Abstract

**Introduction:**

Wheat is a major food crop used in producing bread, noodles, and cookies. Kernel vitreousness, closely related to protein content, is key to determining wheat’s processing purpose. Traditionally, vitreousness is visually assessed, but studies on classifying vitreous and starchy kernels to improve quality are limited.

**Methods:**

This study expands the use of a commercial color sorter to classify kernel vitreousness by G value, distinguishing vitreous from starchy kernels.

**Results and Discussion:**

The system improved protein content and bread-making quality by classifying vitreous kernels, while reducing variability across 23 samples collected over four years. An industrial field test confirmed its applicability at scale. Genetic and environmental factors were also examined, revealing that varietal differences and flowering time were not significant contributors to variations in vitreousness. The findings suggest that color sorting is a reliable tool for enhancing wheat quality until more environmentally stable cultivars are developed, providing economic benefits through improved and consistent product quality.

## 1 Introduction

Wheat (*Triticum aestivum* L.) is a highly important cereal crop globally and accounts for 20% of calories and proteins in human diet (Erenstein et al., 2022; Shewry and Hey, 2015). Wheat is milled into flour and used for diverse processing for producing breads, noodles, cookies, and cakes (Vancini et al., 2019). Several end-use traits in wheat are classified into four categories: grain characteristics, milling properties, flour and dough properties, and baking qualities (Subedi et al., 2023). Grain characteristics include protein content, kernel color, kernel weight, and grain hardness, while milling properties cover flour yield, protein and moisture content, and ash content. Flour and dough properties encompass starch content, falling number, gluten characteristics, and dough rheology. Baking qualities include loaf height, volume, texture, elongation, mixing time, and baking score. Among these traits, grain characteristics are considered a critical quality factor for both classifying and predicting wheat quality. This importance stems from the fact that grain characteristics can be assessed using smaller sample sizes compared to the evaluation of flour and dough properties or baking qualities (Dowell, 2000; Hayes et al., 2017). Therefore, the classification of kernels based on several criteria, such as hardness, vitreousness, and protein content, is a very important step in determining the final end-use quality of wheat (Habernicht et al., 2002).

Kernel vitreousness is a highly important and widely used factor in grading and marketing hard wheat, because it enables the prediction of milling properties and baking quality without destroying the grains (Baasandorj et al., 2015; Dexter and Edwards, 1998). In the US, hard red spring wheat has three subclasses determined by vitreous kernel rate, while the Canadian Grain Commission classifies Canadian Western red spring wheat into three grades by kernel vitreousness (Dexter et al., 2006; Lukow, 2006). In contrast, hard red winter in wheat in US does not have any subclasses, nor do Korean hard red and hard white wheats have such classifications (Lukow, 2006; Lee et al., 2002). Kernel vitreousness is highly correlated with the protein content, hardness, milling properties, and baking quality (Baasandorj et al., 2016; Konopka et al., 2015; Wang et al., 2002). Hard vitreous kernels have relatively high hardness and protein content, resulting in high amount of damaged starch and gluten for excellent baking absorption and improved loaf volume (Liu et al., 2014).

Wheat kernel vitreousness and protein content are highly influenced by environmental variables, including the year and region of cultivation, even within the same hard wheat cultivars (Horrobin et al., 2003; Rozbicki et al., 2015; Williams et al., 2008). The current standard method for evaluating the percentage of vitreous kernels involves manual inspection of a 15 g sample (USDA, 1997). Vitreous kernels have a glasslike and translucent endosperm, while non-vitreous (hereafter referred to as “starchy”) kernels are white, mealy, and light-colored opaque (Baasandorj et al., 2016). As the inspectors’ subjectivity can influence the assessment of kernel vitreousness, several methods for determining kernel vitreousness have been developed using machine vision, color digital cameras, and visible and near-infrared spectroscopy (Gorretta et al., 2006; Symons et al., 2003; Venora et al., 2009). Optimum wavelengths have also been identified to classify vitreous kernels using visible and near-infrared spectroscopy (Wang et al., 2002). These methods enable the estimation of kernel vitreousness without subjectivity. However, studies on large-scale and rapid sorting systems for classifying vitreous and starchy kernels before end-use are scanty.

In several agricultural products, color sorters are used for detecting and separating good-quality grains from poor-quality grains, based on color differences (Lee et al., 2020). Color sorters generally consist of feed systems, optical systems, ejection processes, and image-processing algorithms (Bee & Honeywood, 2007). The optical system measures the reflectivity of each grain, and the ejection system removes unwanted products using compressed air. Color sorters have been used to improve the commercial value of rice, soybeans, peas, and peanuts (Inamdar and Suresh, 2014; Pasikatan and Dowell, 2003). Color sorter-based devices have been developed and tested for separating red wheat from white wheat post-harvest (Pasikatan and Dowell, 2003; Pearson, 2010). Furthermore, a color sorter has been used to select the kernel color within segregating wheat-breeding populations (Brabec et al., 2017). However, color sorters have not been used to separate vitreous kernels from starchy kernels for improving the quality of hard wheat.

Here, we aimed to challenge the traditional use of color sorters by using them to separate vitreous kernels from starchy kernels in large volumes, thereby providing a new perspective on their applications. We sought to determine whether the color sorter could distinguish kernel vitreousness for analyzing the differences in kernel protein contents that affects bread-making quality. We aimed to establish a color-sorting system for enabling mass and rapid classification of vitreous kernels and assessing its effect on reducing quality variation and improving the bread-making quality of hard wheat. Additionally, we aimed to identify the factors causing differences in kernel vitreousness within the same cultivars cultivated in the same field, using both genetic and environmental approaches.

## 2 Materials and methods

### 2.1 Wheat grain samples

Annual and regional wheat grain samples were collected and analyzed, as summarized in **Table 1**. Initially, three hard white wheat cultivars, Keumgang, Jokyoung, and Baekkang, were collected from Sacheon-si in 2019 and used for hand-sorting and machine-sorting tests. After setting up the machine-sorting conditions, Korean hard white wheat cultivars (Keumgang, Jokyoung, and Baekkang) and hard red wheat cultivar Hwanggeumal were collected over 4 years (2020–2023) and analyzed to determine the effectiveness of the machine-sorting system. Wheat samples were collected from two regions (Gwangju and Sacheon-si) in 2020 and 2021, eight regions (Gwangju, Sacheon-si, Hampyeong-gun, Gurye-gun, Jinju-si, Buan-gun, Gimje-si, and Asan-si) in 2022, and three regions (Sacheon-si, Gokseong-gun, and Jeongeup-si) in 2023. The hard white wheat cv. Keumgang was collected from Gwangju and Gurye-gun, while cv. Jokyoung was collected from Sacheon-si and Gurye-gun. Hard white wheat cv. Baekkang, the recommended hard wheat cultivar in Korea, was the most widely collected, with samples from all eight regions. The hard red wheat cv. Hwanggeumal was collected from six regions: Sacheon-si, Hampyeong-gun, Gimje-si, Asan-si, Gokseong-gun, and Jeongeup-si. All these cultivars are the major hard wheat varieties cultivated in the Republic of Korea. cv. Baekkang and cv. Hwangeumal, collected from Jeongup-si in 2023, were also used for a field demonstration test of the machine-sorting system.

**Table 1:** Summary of wheat grain samples used in this study.

Year	Region	Cultivar	
		White wheat	Red wheat
2019	Sacheon-si	Keumgang, Jokyoung, Baekkang	
2020	Gwangju	Keumgang	
	Sacheon-si	Jokyoung	
2021	Gwangju	Keumgang	
	Sacheon-si	Baekkang, Jokyoung	Hwanggeumal
2022	Gwangju	Baekkang	
	Sacheon-si	Baekkang	Hwanggeumal
	Hampyeong-gun	Baekkang	Hwanggeumal
	Gurye-gun	Keumgang, Jokyoung	
	Jinju-si	Baekkang	
	Buan-gun	Baekkang	
	Gimje-si		Hwanggeumal
	Asan-si		Hwanggeumal
2023	Sacheon-si	Baekkang	Hwanggeumal
	Gokseong-gun	Baekkang	Hwanggeumal
	Jeongeup-si	Baekkang	Hwanggeumal

### 2.2 Classification of wheat grains by vitreousness

#### 2.2.1 Hand sorting

**Figure 1:**
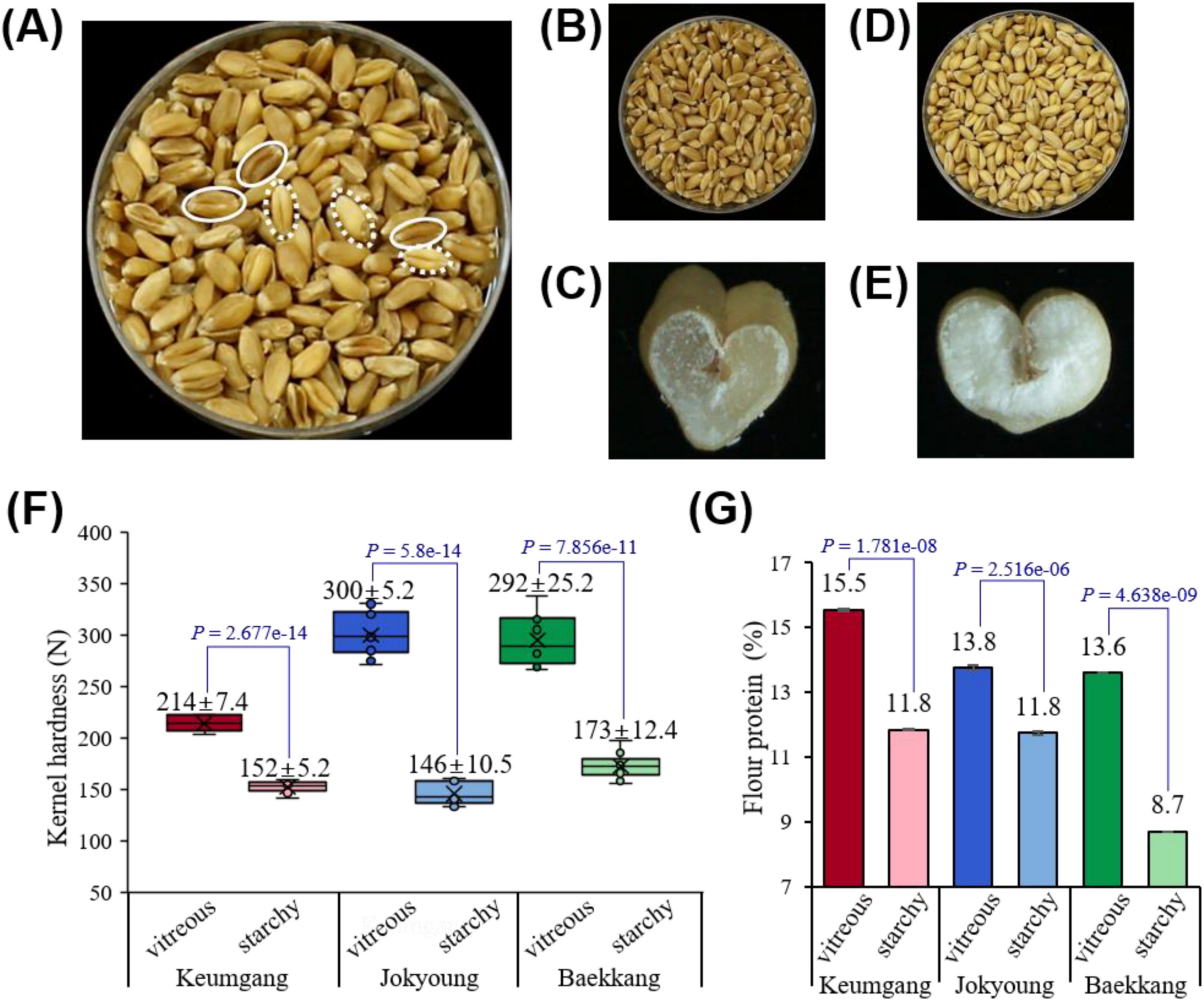
Differences in kernel vitreousness in relation to kernel hardness and protein content. **(A)** Mixed vitreous and starchy kernels observed in Korean hard white wheat cv. Baekkang. The solid circle represents vitreous kernels, while the dashed circle represents starchy kernels. **(B, C)** Hand-sorted vitreous kernels. **(D, E)** Hand-sorted starchy kernels. **(F)** Significant differences in kernel hardness between vitreous and starchy kernels (*P* < 0.0001). **(G)** Significant differences in flour protein contents between vitreous and starchy kernels (*P* < 0.0001).

Wheat kernels were hand-sorted by sensory evaluation (**Figure 1A**). Kernels with a dark color and hyaline cross-section were classified as the vitreous group (**Figures 1B, C**), whereas kernels with a light color and opaque endosperm were classified as the starchy group (**Figures 1D, E**).

#### 2.2.2 Machine sorting

To enable mass and automatic classification by kernel vitreousness, wheat kernels were classified using a SPARK color sorter (DAEWON GSI Co., Chilgok, Republic of Korea) equipped with a 64-channel color camera. The red (R), green (G), and blue (B) color values and speed of kernel input were adjusted until the vitreous and starchy kernels were visually distinct. An RGB converter program (DAWON GSI Co.) was used to select the color values for sorting wheat kernels.

For field demonstration, a SAPARK IA + color sorter (DAEWON GSI Co., Chilgok, Republic of Korea) equipped with an 84-channel color camera was installed at a grain company in Jeongeup-si. The G values were set to 50 and 47 for 1^st^ and 2^nd^ sorting, respectively. The supply amount was 25% for both sorting.

### 2.3 Evaluation of grain quality

#### 2.3.1 Assessment of kernel color

Kernel lightness (L), red/green value (a), and blue/yellow value (b) were measured using a spectrophotometer (CM-3500d; Konica Minolta, Tokyo, Japan), following the manufacturer’s instructions. The RGB values of kernel were extracted using EzPhoto3 program (Hancom Inc., Seongnam, Republic of Korea).

#### 2.3.2 Kernel hardness

Kernel hardness was measured using a DuraVision macrohardness tester (ZwickRoell GmbH & Co. KG, Ulm, Germany). Ten to 20 kernel samples from each classification were tested to determine the average normal force.

#### 2.3.3 Agronomic traits

To identify the agronomic traits of kernel, test weight (TW) was measured using a Graintec Scientific test weight kit (Graintec Scientific, Toowoomba, Australia). Thousand-grain weight (TGW) was measured according to the Rural Development Administration (RDA) Standard Evaluation Manual for Agricultural Experiments and Research (RDA, 2012). TW and TGW were measured in thrice for each sample.

### 2.4 Evaluation of flour quality

#### 2.4.1 Milling and milling properties

Following the American Association of Cereal Chemists (AACC) international experimental milling method 26-50.01 (International, 2010), all wheat samples were conditioned to 15% moisture content and milled with a Brabender Quadurmat Junior mill (CW Brabender Instruments, INC., South Hackensack, NJ, USA). To test bread-making quality, hard white wheat cv. Baekkang collected from Sacheon-si in 2022 was milled using a Buhler MLU 202 laboratory mill (Bühler AG, Uzwil, Switzerland), following the Bühler method for hard wheat 26-21.02 (International, 2010).

After milling, flour protein content was determined using LECO FP628 (Laboratory Equipment Co., St. Joseph, MI, USA), applying a modification factor of 5.7. The ash content was measured following AACC method 08-01.01.

#### 2.4.2 Flour and dough properties and baking qualities

For evaluating flour and dough properties, the gluten content and index were tested using Glutomatic 2200 (Perten Instruments AB, Sweden) following the AACC method 38-12.02 (International, 2010). The falling number were measured following AACC method 56-81.04. The sodium dodecyl sulfate (SDS) sedimentation test was conducted to evaluate both the quantity and quality of wheat flour protein, following the method of Axford and Mcdermott (1979) with a 3 g sample. All results were standardized based on 14% moisture content.

Bread-making quality was evaluated following the AACC method 10-10.03. Ingredients without any additional formula were fermented with 5.3% yeast for 90 min.

### 2.5 Causes of vitreous and starchy grains

#### 2.5.1 Identification of environmental effects

To identify the effects of flowering time and maturity period on kernel vitreousness, two hard white wheat cultivars (Keumgang and Jokyoung) and one hard red wheat cultivar (Hwanggeumal) were sown in early November 2020 in a field with a spacing of 30 × 15 cm (one seed per hill) in the experimental field of the National Institute of Crop Science, RDA, Miryang. A fertilizer consisting of 91 kg N, 74 kg P_2_O_5_, and 39 kg K_2_O per hectare was applied. The stem of each cultivar was tagged when the ear emerged completely above the flag-leaf ligule in April 2022. From the time the first ear emerged, tagging was performed four times, according to the time when the ear emerged from each tiller of each cultivar. Each tagged spike was divided into upper, middle, and bottom sections and threshed for evaluating agronomic and quality traits.

#### 2.5.2 Distinction of genetic background

A single nucleotide polymorphism-based test kit, MSM35-T200, was used for analyzing genetic variation in each cultivar (Misogene, Daejeon, Republic of Korea). This kit enables differentiation of Korean cultivars using allele-specific polymerase chain reaction and multiplex polymerase chain reaction. DNA sample preparation, polymerase chain reaction (PCR), agarose gel electrophoresis, and data analysis were performed according to the manufacturer’s instructions. PCR and agarose gel electrophoresis were performed as follows: initial denaturation at 95°C for 15 min; 32 cycles of 95°C for 20 s, 60°C for 40 s, and 72°C for 40 s; followed by electrophoresis of the PCR products on a 3% agarose gel. The sizes of the PCR products were used to distinguish cultivars.

### 2.6 Statistical analysis

All statistical analyses, including analysis of variance, Duncan’s multiple range test, and *t*-test, were conducted using SAS Enterprise Guide v.7.13 (SAS Institute Inc., Cary, NC, USA). Principal component analysis (PCA) and network plot analysis were performed using RStudio v.1.4.1717 (RStudio, PBC, Boston, MA, USA).

## 3 Results

### 3.1 Grain characteristics vary with kernel vitreousness

The color difference between each group was distinguishable by both the naked eye and graphical color space. Vitreous kernels showed significantly lower R, G, B, L, and b values compared to starchy kernels, while no significant difference was observed in the a value (**Supplementary Tables S1, S2**). Kernel hardness and flour protein content were significantly higher in the vitreous group. The hardness of vitreous kernels was greater across all three hard white wheat cultivars – Keumgang, Jokyoung, and Baekkang – measuring 214 N, 300 N, and 292 N, respectively. In contrast, the hardness of starchy kernels for these cultivars was 152 N, 146 N, and 173 N, respectively (**Figure 1F**). The flour protein content of vitreous kernels was 2.0–4.9% higher in all three cultivars than that of starchy kernels (**Figure 1G**).

### 3.2 Classification of kernel protein content using the color sorter

#### 3.2.1 Setting the color sorter to distinguish kernels by vitreousness

**Figure 2:**
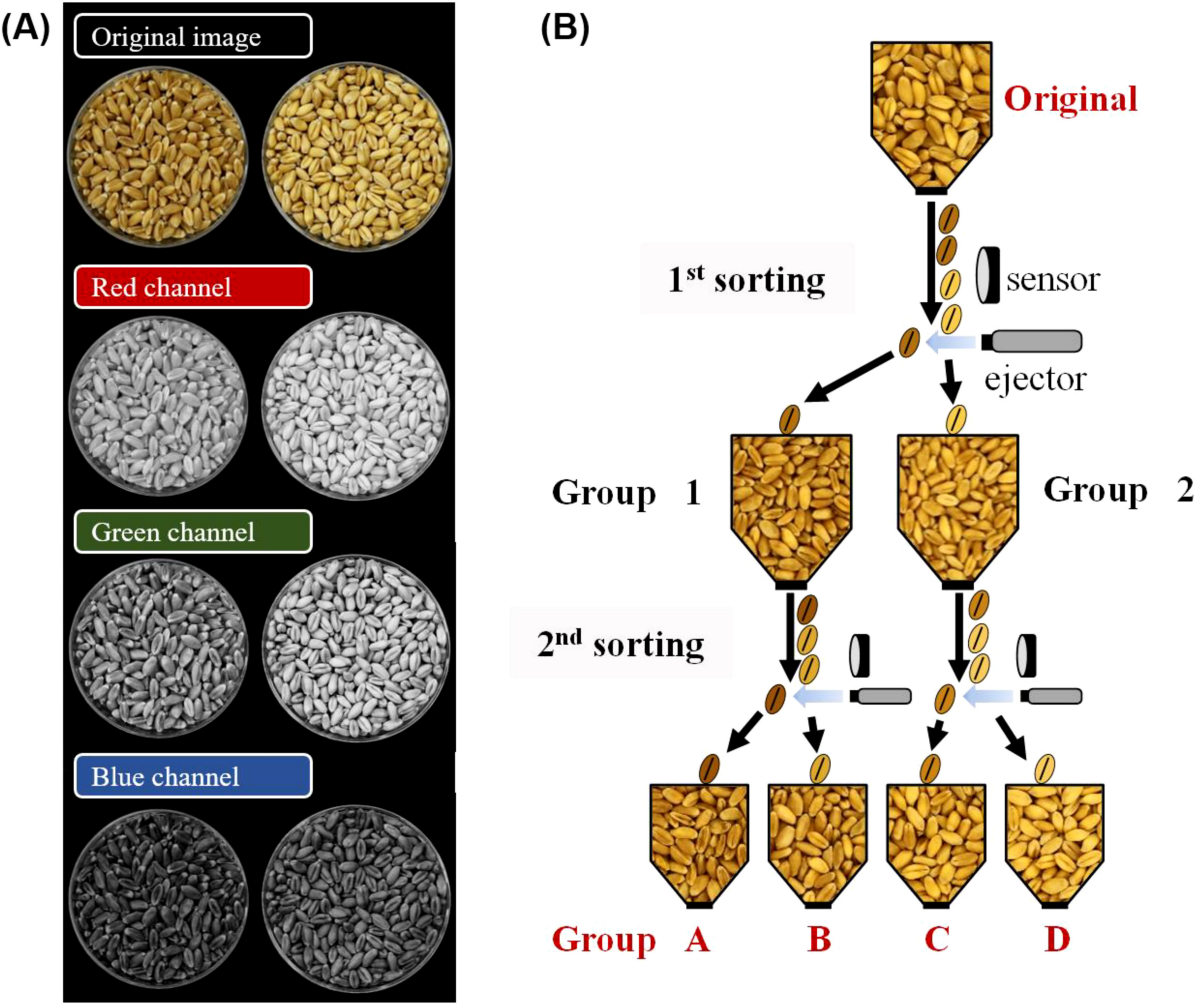
Classification of vitreous kernels from starchy kernels using a color sorter. **(A)** An image of hard white wheat cv. Keumgang from an RGB converter program. **(B)** A diagram of classifying wheat kernels into four groups based on kernel vitreousness, using a color sorter.

As color sorters distinguish colors and materials by their R, G, and B values, an image containing hand-sorted vitreous and starchy kernels was converted using an RGB converter program. Among the R, G, and B channels, the G channel was identified as best for distinguishing between vitreous and starchy kernels (**Figure 2A**). The G values of the color sorter were adjusted until the two groups of kernels were highly distinguishable by the naked eye. Kernels of the three hard wheat cultivars were classified three times to maximize the distinction (**Figure 2B**). The first classification was conducted with a G value of 31 and supply amount of 10%, resulting in groups 1 and 2. Then, group 1 was classified into groups A and B, with a G value of 29 and supply amount of 1%. Group 2 was classified into groups C and D, with a G value of 39 and supply amount of 1%.

**Figure 3:**
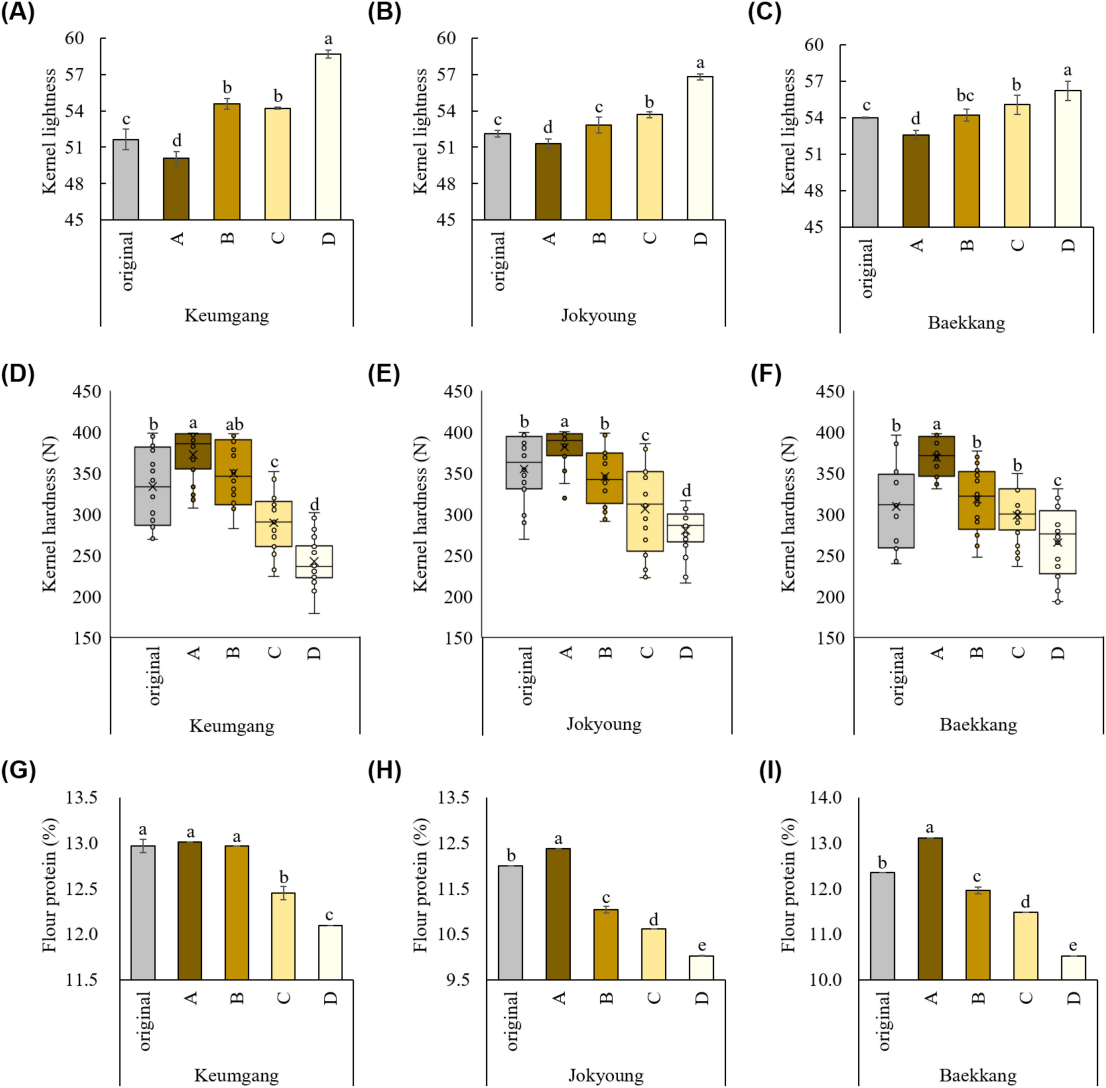
Different kernel traits between groups classified with the color-sorting system. Difference in kernel lightness among **(A)** cv. Keumgang, **(B)** cv. Jokyoung, and **(C)** cv. Baekkang. Difference in kernel hardness among (D) cv. Keumgang, **(E)** cv. Jokyoung, and **(F)** cv. Baekkang. Difference in flour protein contents between groups in **(G)** cv. Keumgang, **(H)** cv. Jokyoung, and **(I)** cv. Baekkang. Different letters on plots indicate significant differences between each other (*P* <0.01).

Group A showed the darkest color, whereas group D showed the lightest kernel color, with significant differences in L and b values, consistent with the hand-sorting experimental results (**Figures 3A–C**, **Supplementary Table S3**). No significant difference in the a value was observed among groups in cv. Jokyoung and cv. Baekkang, whereas cv. Keumgang exhibited a significantly lower a value in group D compared to the other groups. A comprised 52.5–83.4% of the total sorted kernels, whereas group D comprised 2.0–8.2% (**Supplementary Table S4**). Kernel hardness was significantly higher in group A while significantly lower in group D (**Figure 3D–F**). The average kernel hardness values of group A were 372, 381, and 368 N for hard white wheat cultivars Keumgang, Jokyoung, and Baekkang, respectively. These values were 7–16% higher than those of original kernels and 26–35% higher than those of kernels in group D. The standard deviation in kernel hardness was also significantly lower in groups A and D compared to the original samples, indicating that kernel quality achieved greater uniformity as a result of the color-sorting process. The flour protein content was also significantly higher in group A than in group D. cv. Keumgang, cv. Jokyoung, and cv. Baekkang showed 0.9%, 2.4%, and 2.6% higher flour protein contents in group A than those in group D, respectively (**Figure 3G–I**). cv. Jokyoung and cv. Baekkang also showed 0.4–0.7% increase in flour protein content in group A compared to that of the original kernels. The SDS sedimentation value was higher in group A than in group D (**Supplementary Table S5**). No significant difference was observed in the falling number that indicates α-amylase activity. Ash content did not exhibit a consistent trend across groups, with the highest values recorded in groups A and B for cv. Keumgang, groups A, B, and C for cv. Jokyoung, and groups B and D for cv. Baekkang. Thousand-grain weight was significantly higher in groups B and C than in groups A and D for all three cultivars (**Supplementary Table S4**). No significant differences in test weights were observed among the groups in cv. Baekkang, However, group D exhibited the lowest test weight among the groups in both cv. Keumgang and cv. Jokyoung.

#### 3.2.2 Determination of wide range of effectiveness of the machine-sorting system

**Figure 4:**
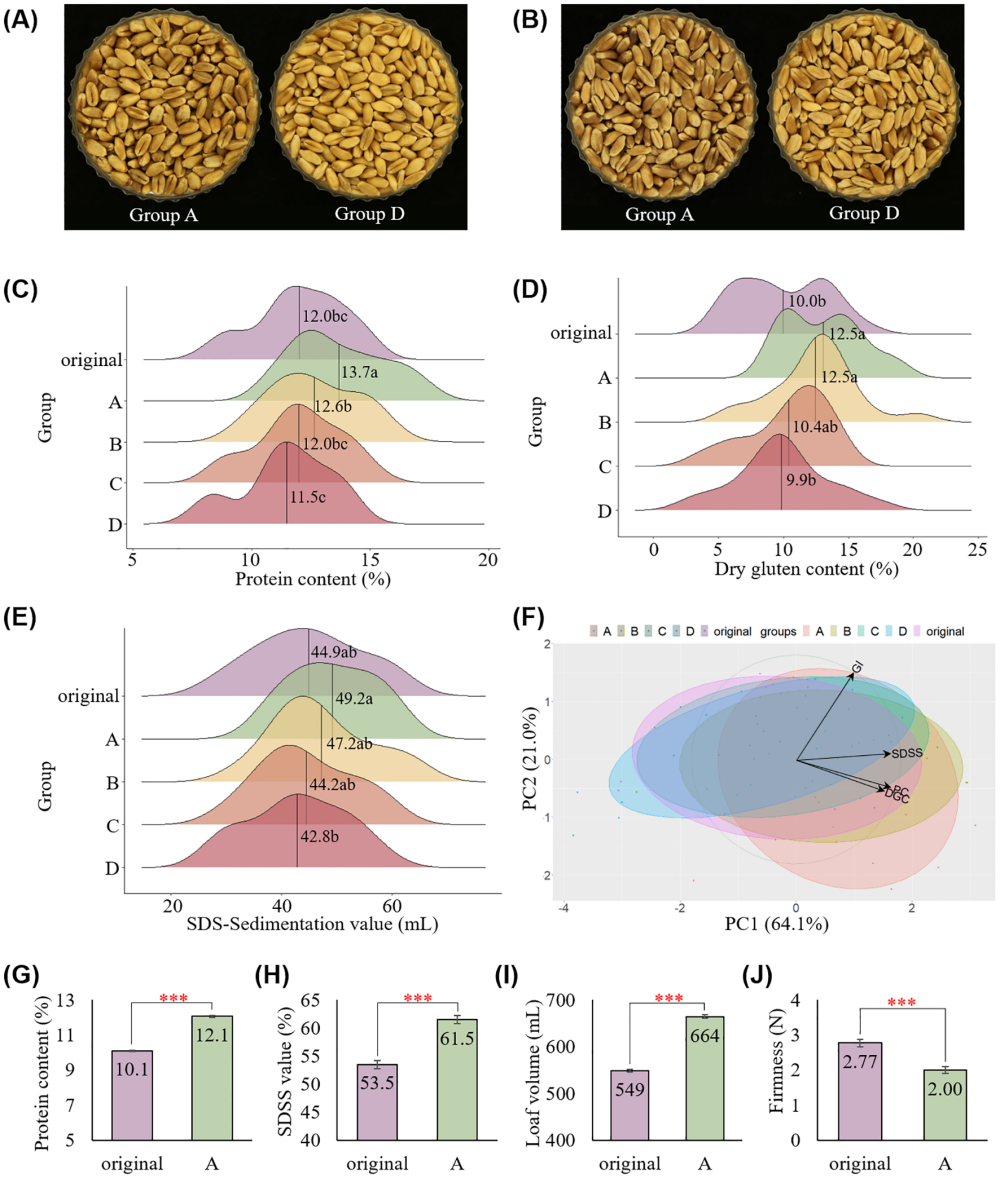
Application of the color-sorting system on 23 wheat samples collected from 10 regions in Korea over four years. Different kernel colors observed between groups in **(A)** hard white wheat cv. Jokyoung and **(B)** hard red wheat cv. Hwanggeuaml. Variation in **(C)** protein content, **(D)** dry gluten content, and **(E)** SDS sedimentation values among groups. Different letters on the plots indicate significant differences (*P* < 0.01). **(F)** PCA analysis on protein content (PC), dry gluten content (DGC), gluten index (GI), and SDS-sedimentation value (SDSS) categorized by sorted groups. Increased kernel vitreousness in cv. Baekkang resulted in high **(G)** protein content, **(H)** SDS sedimentation value, **(I)** loaf volume, and **(J)** low bread firmness (*** *P* < 0.001).

To determine the effectiveness of the color-sorting system in distinguishing kernel vitreousness and improving flour quality by reducing deviation among kernels, bread wheat grain samples of four cultivars were collected and analyzed over 4 years (2020–2023), as summarized in **Table 1**. Each group of kernels was visibly different from the others, and groups A and D were very clearly distinguishable (**Figures 4A, B**). The hard red wheat cultivar, Hwangeumal (**Supplementary Figure S1**), was also well classified, with the same G values as those of the hard white wheat cultivars. Wheat protein content showed significant variations depending on cultivation years, regions, and cultivars (Kramer, 1979) (**Supplementary Table S6**). Therefore, we focused on the sorting effect rather than the environmental effects and compared the quality traits by group after sorting. Among the 23 samples tested, group A comprised 4.8–26.9% of the total sorted kernels, while group D comprised 14.1–30.6% (**Supplementary Table S7**).

The flour protein and gluten contents of group A improved by 1.7% and 2.5%, respectively, compared to those of the original sample (**Figures 4C, D**). Consequently, the SDS sedimentation value also improved in group A, with no change in the gluten index (**Figure 4E**, **Supplementary Table S7**). Groups B and C showed quality traits similar to the original samples, whereas group D showed decreased quality traits. These differences in quality between groups were also confirmed by PCA (**Figure 4F**). Notably, the distribution of these quality traits decreased compared to that of the original samples, despite the samples being collected from various environments.

To confirm the positive effect of this color-sorting technique on bread-making quality, hard white wheat cv. Baekkang collected from Sacheon-si in 2022 was milled. In accordance with the previous finding, the protein content and SDS sedimentation value improved in group A compared to those of the original sample (**Figures 4G, H**). Loaf volume and bread firmness were significantly different between the original sample and group A. Group A showed a higher loaf volume and lower bread firmness compared to the original sample, indicating that the final processing quality of bread wheat improved as a result of color sorting (**Figures 4I, J**, **Supplementary Figure S2**).

#### 3.2.3 Field demonstration test of the color-sorting system for kernel vitreousness

**Figure 5:**
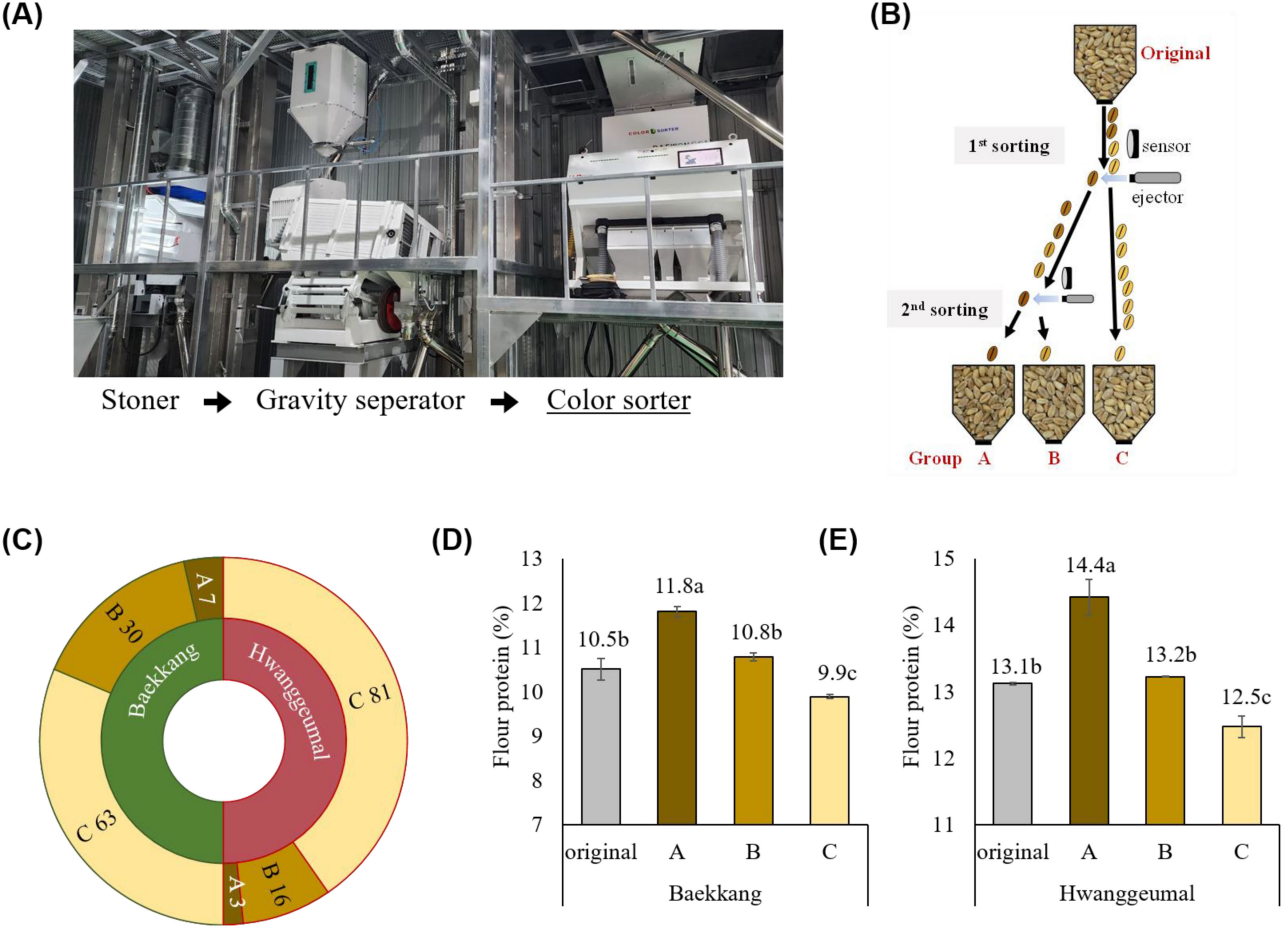
Field demonstration test of the color sorting system in an industrial setting. **(A)** Layout of the grain cleaning and sorting process. **(B)** A simplified diagram of classifying wheat kernels into three groups based on kernel vitreousness, using the color sorter. **(C)** Proportion of each grade in hard white wheat cv. Baekkang and hard red wheat cv. Hwanggeumal. Differences in protein contents among groups in **(D)** cv. Baekkang and **(E)** cv. Hwanggeumal (*P* < 0.01).

A field demonstration test was conducted in an industrial setting to assess the efficiency of the color-sorting system. A color sorter was installed next to a stoner and a gravity separator, enabling stepwise selection before storage and milling (**Figure 5A**). To simplify grading, kernels were classified into three groups (**Figure 5B**). The first sorting step was performed when the original kernels were supplied through a hopper. The relatively light-colored kernels were immediately classified as group C. The relatively dark-colored kernels were ejected back into the hopper for a second sorting and then classified into groups A and B. The sorting speed was approximately 2 ton/h for both cv. Baekkang and cv. Hwanggeumal. The distribution in each group was 7% A, 30% B, and 63% C in cv. Baekkang, and 3% A, 16% B, and 81% C in cv. Hwanggeumal (**Figure 5C**). Flour protein content improved by 1.3% in group A for both cultivars compared to that of the original kernels (**Figures 5D, E**). Group C showed a significantly lower protein content (0.7–0.9%) than that of the original kernels.

### 3.3 Reason for the difference in kernel vitreousness

The genetic backgrounds of vitreous and starchy kernels were identified. No significant differences in cultivar purity were observed among the groups examined using molecular markers (**Supplementary Table S8**, **Supplementary Figure S3**).

**Figure 6:**
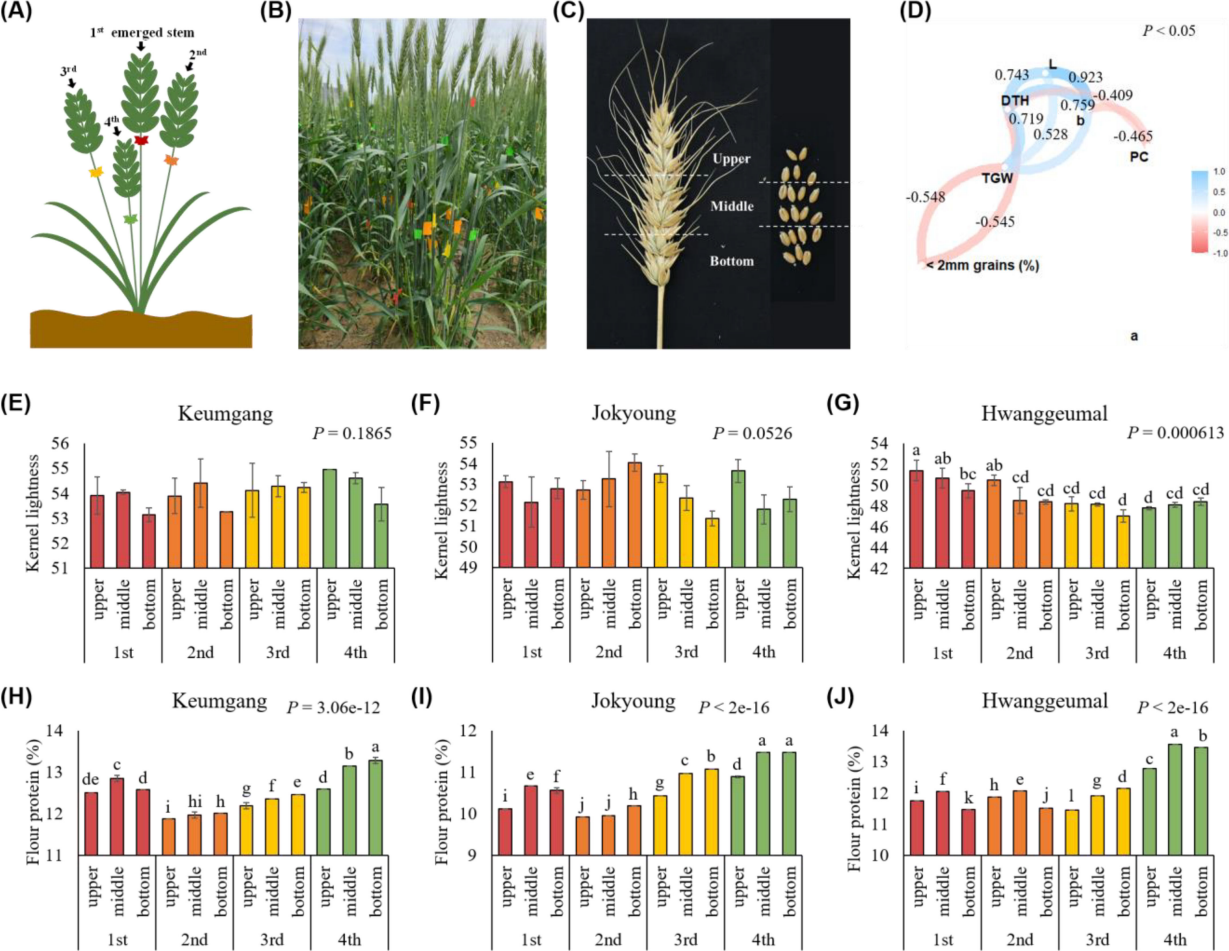
Reason for differences in kernel vitreousness in the same cultivar in the same field according to flowering time. **(A)** Tagging stems according to the heading time. **(B)** Tagging in field conditions. **(C)** Dividing a spike into three sections. **(D)** Network plot of a correlation between grain characteristics and protein content. L: kernel lightness; DTH: days to heading; b: b value; PC: flour protein content; TGW: thousand-grain-weight. Differences in kernel lightness between tillers and spikelet order in **(E)** cv. Keumgang, **(F)** cv. Jokyoung, and **(G)** cv. Hwanggeumal. Differences in flour protein contents between tillers and spikelets order in **(H)** cv. Keumgang, **(I)** cv. Jokyoung, and **(J)** cv. Hwanggeumal. Different letters indicate significant difference (*P* < 0.01).

To analyze the cause of the differences in vitreousness and protein content in the same cultivar cultivated in the same field, wheat stems were tagged and harvested separately according to heading time (**Figures 6A, B**). Each spike was then separated into three parts (upper, middle, and bottom) and threshed to evaluate traits related to kernel vitreousness (**Figure 6C**). Kernel lightness (L) was highly correlated with days from heading to harvest (DTH) and b value (b) (**Figure 6D**). Kernel lightness also correlated with TGW; however, no significant correlation was observed between kernel lightness and flour protein content (PC). Protein content was negatively correlated with DTH and b value.

When each part of the spike of each cultivar was analyzed, no significant differences in kernel lightness between spikelet positions were observed in cv. Keumgang and cv. Jokyoung, whereas kernels from the upper part of the spike from the first emerged stem showed the highest kernel lightness in cv. Hwanggeumal (**Figures 6E–G**). Flour protein content was highest in the middle or bottom parts of the latest emerged spikes (4^th^ spike) across all three cultivars in this study. (**Figures 6H–J**).

## 4 Discussion

### 4.1 A new role of the color sorter in classifying kernel vitreousness

Hard and soft wheat are classified based on variations in the *Puroindoline a* (*Pina-D1*) and *Puroindoline b* (*Pinb-D1*) genes located on chromosome 5D in wheat (Mohammadi et al., 2013). The presence of wild-type alleles (*Pina-D1a* and *Pinb-D1a*) results in soft-textured wheat, whereas mutations in either of these genes lead to hard-textured wheat kernels. However, kernel vitreousness and hardness are affected by the environment; hard wheat can become starchy, whereas soft wheat can become vitreous (Pasha et al., 2010). We confirmed that vitreous and hard wheat kernels had protein contents higher than those of starchy and soft wheat kernels, even when they were from the same cultivars and harvested from the same fields, which is in accordance with the findings of previous studies (Baasandorj et al., 2016; Konopka et al., 2015; Liu et al., 2014). This also presents the possibility of improving the wheat grain protein content by sorting based on kernel phenotypes.

Several studies have suggested methods for determining kernel vitreousness using camera-based machines to avoid assessment through the naked eyes (Gorretta et al., 2006; Symons et al., 2003; Venora et al., 2009; Wang et al., 2002). However, studies on classifying and identifying vitreous kernels using a machine-based mass-sorting system are scanty. Our results showed that kernels can be classified by vitreousness using a commercial color sorter. Classification by kernel vitreousness confirmed the changes in quantity and deviation of protein content. End-use quality improved as a result of increased protein content in the vitreous group classified using the color sorter. No significant differences were observed in the gluten index, as shown in **Figure 4F** and **Supplementary Table S7**, indicating that kernel vitreousness increased bread-making quality owing to increased protein quantity, but not quality, which corresponds with the findings of Fu et al. (2018). Tests with 23 wheat samples collected over 4 years confirmed the stability of the color-sorting system. In addition, a field demonstration test confirmed the applicability of this system in an industrial setting.

Each color sorter can have different equipment, including cameras, sensors, and operating programs, depending on their production time, reflecting ongoing advancements in technology (Jivani et al., 2024). Therefore, experimental conditions were set in this study; however, the G value and supply amount were adjusted until the kernels were distinguishable by the naked eye. Two grouping methods were evaluated: a four-group classification (A–D) for development and assessment of the color sorting methods and a three-group classification (A–C) for field demonstration. The key point of this study is that kernels in large volumes can be classified into high- and low-protein content groups using the color sorter. Additionally, the selection of sorting methods, whether using groups A–D, A–C, or other approaches, depends on the specific objectives and preferences of the users. The three-group classification demonstrated in this study was designed to improve the protein content of hard white wheat cv. Baekkang for enhancing bread-making quality, rather than to further subdivide low-protein-content groups.

Using the color-sorting technique, wheat can be categorized into groups with different protein contents, thereby making it suitable for various processing purposes, as illustrated in a schematic process shown in **Supplementary Figure S4**. The schematic in **Supplementary Figure S4** is based on real experimental data obtained during the development and evaluation of the color-sorting methods in this study, demonstrating their effectiveness in classifying and standardizing wheat kernel quality. The observed variation in the percentage of cross groups in different cultivars suggests that genetic factors may influence the effectiveness of sorting process. Therefore, this system can also be used for selecting bread wheat cultivars for each cultivation area. Furthermore, consistency in improving quality traits despite environmental variations underscores the robustness of the color-sorting system. This consistency could lead to a highly predictable and reliable wheat quality, benefiting both producers and consumers. For example, even within the same bread wheat cultivar, protein content can significantly vary depending on the region and year of production (Triboï et al., 2003). In some cases, protein contents may be too low for bread-making. This color-sorting technique provides a practical approach to mitigating these variations by selectively isolating kernels with adequate protein content, ensuring a more consistent and reliable wheat quality for bread-making.

### 4.2 Factors influencing kernel vitreousness and protein content in wheat

We hypothesized that genetic and environmental factors may cause differences in kernel vitreousness. However, no significant difference was observed in the genetic background between vitreous and starchy kernels, and differences in flowering rate within a single wheat plant, caused by the tillering and flowering order (from the middle portion toward the edges) in the spike, resulted in variations in kernel traits (Tottman, 1987). Xu et al. (2015) have noticed that yield components, such as kernel weight, grain number per spike, and yield per spike, tend to be higher in the main stem than in late-initiated tillers. Pan et al. (2005) and Xuexia et al. (2008) reported variations in grain weight and protein content among different spikelet positions within a spike. Therefore, we hypothesized that the variation in protein content in the same cultivar cultivated in the same field was also owing to different anthesis speeds among spikelets. However, our results indicated that differences in tillering and flowering times caused variations in TGW and kernel lightness but did not directly impact kernel vitreousness and protein content. Short grain-filling periods lead to reduced TGW and increased protein contents because TGW and protein contents are negatively correlated (Kindred et al., 2008; Nagarajan et al., 2007).

Therefore, further research is needed to understand why differences in kernel vitreousness and protein content occur in wheat. Uncovering these factors will greatly contribute to improving wheat quality and stability. In the meantime, the color sorting system we have developed is likely to be one of the most reliable technologies for stabilizing quality until cultivars that are highly stable against environmental factors can be developed.

### 4.3 Limitations and future work for the application of the vitreousness-sorting system

The successful adoption of this color-sorting technology depends largely on its economic feasibility at a commercial scale, which requires further evaluation. The initial investment in equipment and ongoing maintenance costs must be carefully assessed. Additionally, securing trained personnel to manage the system and adjust sorting criteria based on kernel conditions may present challenges to its large-scale implementation.

While the high-protein group is expected to gain economic value, the potential impact on the lower-protein group, which may have reduced protein content compared to the original kernels, should also be considered.

In this study, the color sorter effectively classified hard wheat cultivars, applying the same criteria for white wheat and red wheat. However, variations in sorter specifications and wheat varieties may affect adaptability and misclassification rates, requiring further validation and optimization under diverse conditions.

Despite these limitations, the long-term benefits of improved wheat quality and consistency could outweigh the initial costs, making this technology a promising solution for industrial applications. Future research should focus on refining sorting parameters for different wheat varieties and developing a comprehensive economic assessment to enhance its practical viability.

## 5 Conclusion

Kernel vitreousness is closely associated with the protein content and end-use quality of wheat. Our study expands the use of color sorters and suggests a new method for producing relatively stable and high-quality wheat products, which is crucial for the baking industry. However, its economic feasibility on a commercial scale requires further investigation. Future research should explore the integration of color sorting with other advanced technologies, such as near-infrared spectroscopy, to further refine the sorting process and improve its accuracy. Additionally, understanding the cause of differences in kernel vitreousness and protein content within the same sample is vital for long-term improvement in wheat quality. Overall, the color-sorting system presents a promising approach for improving wheat quality by leveraging the inherent differences in kernel vitreousness. This method has the potential to transform wheat processing practices, leading to better end-products and increased value in the wheat supply chain.

## Data Availability

The original contributions presented in the study are included in the article/**Supplementary Material**. Further inquiries can be directed to the corresponding author.
