# Supplementary material for: Enhancing wheat quality through color sorting: a novel approach for classifying kernels based on vitreousness
Source: Front Plant Sci. 2025 Apr 17;16:1534621. doi: 10.3389/fpls.2025.1534621 (PMC12043495; doi:10.3389/fpls.2025.1534621)
Supplement: Supplementary file 1 [file DataSheet1.docx]

Supplementary Material

Enhancing wheat quality through color sorting: A novel approach for classifying kernels based on vitreousness

Jin-Kyung Cha^1^, Hyeonjin Park^1^, Youngho Kwon^1^, So-Myeong Lee^1^, Jeonghyun Kim^2^, Woo-Jae Kim^1^, Kwangho Park^3^, Woosik Jang^3^, Youngeun Lee^1^, Byung Jun Jin^1^, Kidong Han^3^, Ki-Won Oh^1^, Jong-Hee Lee^1*^

*** Correspondence:** Jong-Hee Lee : [ccriljh@korea.kr](mailto:ccriljh@korea.kr)

# Supplementary Figures and Tables

## Supplementary Figures


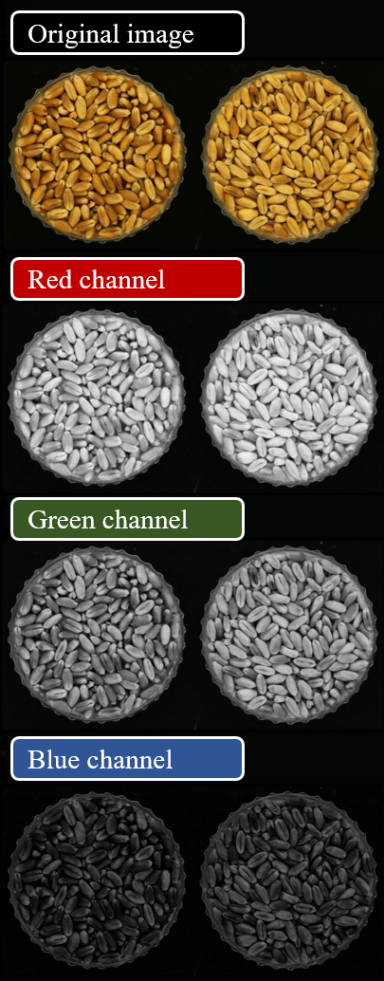


**Fig. S1.** An image of red hard wheat cv. Hwanggeumal from an RGB converter program.


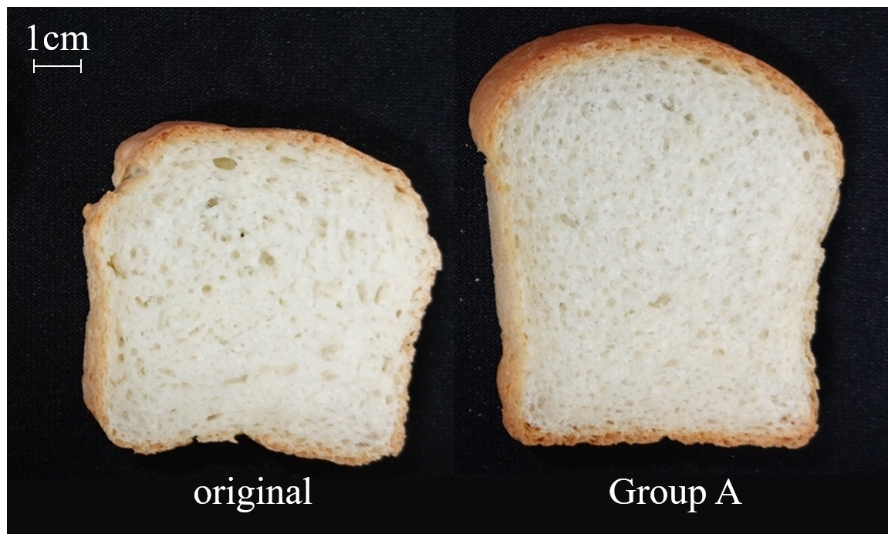


**Fig. S2.** Improved loaf characteristics of cv. Baekkang in group A as a result of color sorting.


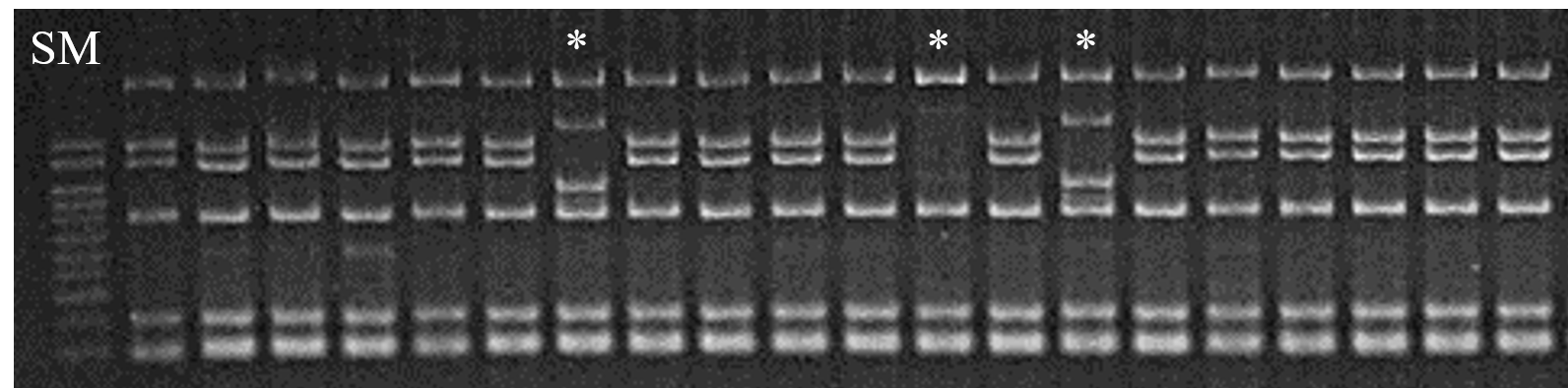


**Fig. S3.** Distinction of genetic variation in each color-sorted group with a molecular marker. SM, standard marker for distinguishing Korean cultivars. *Different cultivar detected from cv. Baekkang.


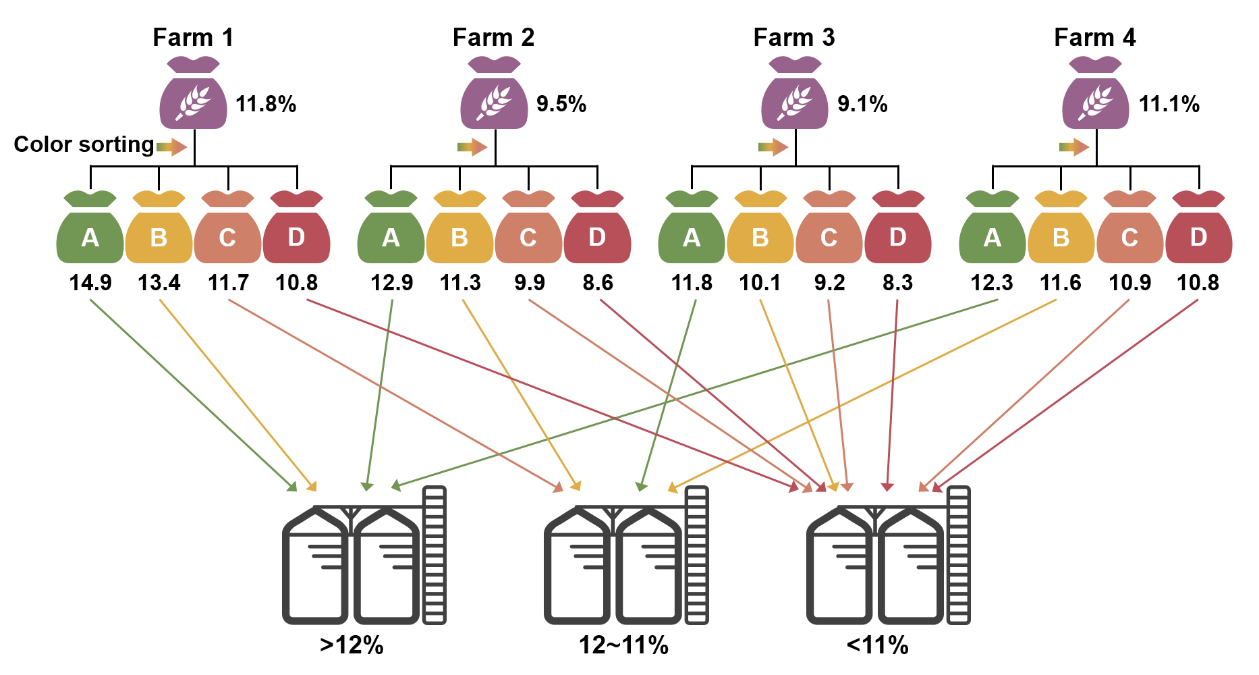


**Fig. S4.** Scheme of the proposed utilization of color sorting system for classified storage of wheat. The color sorter is utilized to classify wheat based on protein content by sorting kernels according to their color, thereby refining the original kernels to better match the target protein content. This schematic is derived from real experimental data obtained in this study.

## Supplementary Tables

**Table S1.** Color differences between hand-sorted vitreous and starchy kernels by color code extracted using EzPhoto3 program (****P* < 0.001).

| **Color difference** | **Keumgang** | | | **Jokyoung** | | | **Baekkang** | | |
| --- | --- | --- | --- | --- | --- | --- | --- | --- | --- |
|  | **vitreous** | **starchy** |  | **vitreous** | **starchy** |  | **vitreous** | **starchy** |  |
| R | 202 | 253 | *** | 184 | 233 | *** | 176 | 247 | *** |
| G | 147 | 211 | *** | 133 | 190 | *** | 118 | 203 | *** |
| B | 49 | 106 | *** | 43 | 91 | *** | 30 | 89 | *** |

**Table S2.** Color difference between hand-sorted vitreous and starchy kernels measured using a spectrophotometer (****P* < 0.001; ns, not significant).

| **Color difference** | **Keumgang** | | | **Jokyoung** | | | **Baekkang** | | |
| --- | --- | --- | --- | --- | --- | --- | --- | --- | --- |
|  | **vitreous** | **starchy** |  | **vitreous** | **starchy** |  | **vitreous** | **starchy** |  |
| L | 47.7 | 54.7 | *** | 47.7 | 54.4 | *** | 47.3 | 57.3 | *** |
| a | 8.2 | 8.3 | ns | 8.0 | 7.9 | ns | 8.8 | 8.7 | ns |
| b | 22.1 | 26.3 | *** | 21.7 | 24.3 | *** | 22.6 | 28.2 | *** |

**Table S3.** Color differences between machine-sorted vitreous and starchy kernels measured using a spectrophotometer. Different letters indicate significant differences from each other (*P* < 0.01; ns, not significant).

| Cultivar | Group | L |  | a |  | b |  |
| --- | --- | --- | --- | --- | --- | --- | --- |
| Keumgang | original | 51.6 | c | 8.8 | a | 23.6 | c |
|  | A | 50.1 | d | 8.8 | a | 22.6 | d |
|  | B | 54.6 | b | 7.9 | b | 24.5 | b |
|  | C | 54.2 | b | 8.3 | ab | 24.4 | b |
|  | D | 58.7 | a | 7.3 | c | 26.8 | a |
| Jokyoung | original | 52.1 | c | 8.7 | ns | 24.4 | bc |
|  | A | 51.3 | d | 8.9 | ns | 24.0 | b |
|  | B | 52.8 | c | 8.4 | ns | 24.3 | b |
|  | C | 53.7 | b | 8.4 | ns | 25.1 | b |
|  | D | 56.8 | a | 8.4 | ns | 27.2 | a |
| Baekkang | original | 54.0 | c | 8.4 | ns | 24.9 | bc |
|  | A | 52.6 | d | 8.4 | ns | 24.2 | c |
|  | B | 54.2 | bc | 8.3 | ns | 24.9 | bc |
|  | C | 55.1 | b | 8.2 | ns | 25.4 | ab |
|  | D | 56.2 | a | 8.2 | ns | 26.1 | a |

**Table S4.** Grain characteristics of each group of sorted kernels. Different letters indicate significant differences from each other (*P* < 0.001; ns, not significant).

| Cultivar | Group | Ratio (%) | Test weight (g/L) | | TGW (g) | | < 2-mm grains (%) |
| --- | --- | --- | --- | --- | --- | --- | --- |
| Keumgang | original | 100.0 | 417 | a | 42.8 | ab | 1.37 |
|  | A | 83.4 | 418 | a | 42.3 | b | 0.71 |
|  | B | 7.6 | 415 | ab | 43.4 | a | 1.10 |
|  | C | 7.0 | 414 | b | 43.5 | a | 0.95 |
|  | D | 2.0 | 411 | c | 40.5 | c | 1.47 |
| Jokyoung | original | 100.0 | 414 | c | 51.7 | b | 0.24 |
|  | A | 70.7 | 418 | b | 51.1 | c | 0.15 |
|  | B | 15.6 | 421 | a | 52.6 | a | 0.11 |
|  | C | 11.1 | 422 | a | 52.1 | ab | 0.16 |
|  | D | 2.6 | 415 | c | 50.5 | c | 0.26 |
| Baekkang | original | 100.0 | 403 | ns | 49.0 | c | 0.60 |
|  | A | 52.5 | 399 | ns | 47.5 | d | 1.57 |
|  | B | 22.1 | 402 | ns | 50.8 | a | 0.63 |
|  | C | 17.3 | 402 | ns | 50.4 | ab | 0.51 |
|  | D | 8.2 | 401 | ns | 49.3 | bc | 0.62 |

**Table S5.** Quality traits of each group of sorted kernels. Different letters indicate significant differences from each other (*P* < 0.001; ns, not significant).

| Cultivar | Group | Protein (%) | | Ash (%) | | Falling Number (s) |  | SDS-sedimentation value (mL) |  |
| --- | --- | --- | --- | --- | --- | --- | --- | --- | --- |
| Keumgang | Original | 13.0 | a | 0.72 | b | 459.5 | ns | 41.2 | a |
|  | A | 13.0 | a | 0.77 | a | 446.0 | ns | 41.2 | a |
|  | B | 13.0 | a | 0.78 | a | 469.5 | ns | 40.5 | a |
|  | C | 12.5 | b | 0.61 | c | 457.5 | ns | 38.6 | b |
|  | D | 12.1 | c | 0.57 | d | 421.0 | ns | 36.1 | c |
| Jokyoung | Original | 12.0 | b | 0.58 | ab | 449.5 | ns | 41.7 | b |
|  | A | 12.4 | a | 0.59 | a | 441.5 | ns | 44.3 | a |
|  | B | 11.0 | c | 0.60 | a | 421.5 | ns | 38.1 | c |
|  | C | 10.6 | d | 0.60 | a | 429.5 | ns | 36.9 | d |
|  | D | 10.0 | e | 0.57 | b | 424.0 | ns | 33.3 | e |
| Baekkang | Original | 12.4 | b | 0.53 | b | 450.5 | ns | 40.5 | c |
|  | A | 13.1 | a | 0.54 | b | 471.0 | ns | 49.3 | a |
|  | B | 12.0 | c | 0.59 | a | 427.0 | ns | 43.9 | b |
|  | C | 11.5 | d | 0.53 | b | 436.0 | ns | 42.7 | b |
|  | D | 10.5 | e | 0.61 | a | 447.0 | ns | 36.9 | d |

**Table S6.** Analysis of variance in protein contents depending on production years, regions, cultivars, and color-sorted groups; Df, degree of freedom; Sum Sq, sum of squares; Mean Sq, mean of squares; Pr, probability.

| **Factor** | **Df** | **Sum Sq** | **Mean Sq** | **F value** | **Pr(>F)** |
| --- | --- | --- | --- | --- | --- |
| Year | 3 | 33.72 | 11.24 | 7.14 | 0.0002*** |
| Region | 9 | 98.20 | 10.91 | 6.93 | 0.0000*** |
| Cultivar | 3 | 81.54 | 27.18 | 17.26 | 0.0000*** |
| Group | 4 | 64.23 | 16.06 | 10.20 | 0.0000*** |
| Residuals | 89 | 140.16 | 1.57 |  |  |

**Table S7.** Application of the color-sorting system on 23 wheat samples collected over 4 years. Different letters indicate significant differences from each other (*P* < 0.05; ns, not significant).

| Cultivar | Group | No. of samples | Percentage (%) | | Protein content (%) | Dry gluten (%) | | | | Gluten index | | SDSS (mL) | |
| --- | --- | --- | --- | --- | --- | --- | --- | --- | --- | --- | --- | --- | --- |
| Keumgang | original | 3 | 100.0 |  | 12.6 | |  | 11.4 |  | 81.3 |  | 50.0 |  |
|  | A |  | 25.1 |  | 13.3 | |  | 12.9 |  | 83.4 |  | 56.5 |  |
|  | B |  | 36.2 |  | 12.5 | |  | 11.8 |  | 85.3 |  | 49.0 |  |
|  | C |  | 20.9 |  | 12.3 | |  | 12.6 |  | 81.8 |  | 50.5 |  |
|  | D |  | 17.8 |  | 12.0 | |  | 12.2 |  | 75.9 |  | 49.5 |  |
| Jokyoung | original | 3 | 100.0 |  | 9.9 | |  | 6.6 |  | 53.9 |  | 32.5 |  |
|  | A |  | 7.4 |  | 13.5 | |  | 11.9 |  | 62.8 |  | 53.0 |  |
|  | B |  | 18.9 |  | 11.7 | |  | 12.1 |  | 77.3 |  | 49.0 |  |
|  | C |  | 43.1 |  | 10.1 | |  | 7.9 |  | 60.8 |  | 39.0 |  |
|  | D |  | 30.6 |  | 9.1 | |  | 6.5 |  | 60.9 |  | 31.0 |  |
| Baekkang | original | 9 | 100.0 |  | 12.2 | |  | 10.4 |  | 77.7 |  | 47.0 |  |
|  | A |  | 4.8 |  | 14.7 | |  | 13.0 |  | 78.0 |  | 51.6 |  |
|  | B |  | 11.8 |  | 13.3 | |  | 12.3 |  | 79.4 |  | 50.6 |  |
|  | C |  | 61.7 |  | 12.4 | |  | 10.3 |  | 82.1 |  | 46.4 |  |
|  | D |  | 21.8 |  | 11.7 | |  | 10.4 |  | 80.7 |  | 44.1 |  |
| Hwanggeumal | original | 8 | 100.0 |  | 12.4 | |  | 10.2 |  | 85.0 |  | 44.6 |  |
|  | A |  | 26.9 |  | 13.2 | |  | 12.2 |  | 84.9 |  | 46.3 |  |
|  | B |  | 24.8 |  | 12.5 | |  | 13.0 |  | 80.2 |  | 44.6 |  |
|  | C |  | 34.2 |  | 12.1 | |  | 10.7 |  | 84.6 |  | 43.3 |  |
|  | D |  | 14.1 |  | 11.9 | |  | 9.7 |  | 82.4 |  | 42.6 |  |
| Total | original | 23 | 100.0 | a | 12.0 | | bc | 10.0 | b | 77.6 | ns | 44.9 | ab |
|  | A |  | 17.1 | c | 13.7 | | a | 12.5 | a | 79.1 | ns | 49.2 | a |
|  | B |  | 21.7 | c | 12.6 | | b | 12.5 | a | 80.3 | ns | 47.2 | ab |
|  | C |  | 41.8 | b | 12.0 | | bc | 10.4 | ab | 80.2 | ns | 44.5 | ab |
|  | D |  | 19.4 | c | 11.5 | | c | 9.9 | b | 78.1 | ns | 42.8 | b |

**Table S8.** Comparison of cultivar purity between groups classified with the color-sorting system. No significant difference was observed between groups (*P* > 0.9546).

| Cultivar | Group | No. of tested seeds | No. of different cultivars | Cultivar purity (%) |
| --- | --- | --- | --- | --- |
| Keumgang | original | 20 | 1 | 95.0 |
|  | A | 20 | 2 | 90.0 |
|  | B | 20 | 0 | 100.0 |
|  | C | 20 | 1 | 95.0 |
|  | D | 20 | 2 | 90.0 |
| Jokyoung | original | 20 | 0 | 100.0 |
|  | A | 20 | 2 | 90.0 |
|  | B | 20 | 1 | 95.0 |
|  | C | 20 | 1 | 95.0 |
|  | D | 20 | 1 | 95.0 |
| Baekkang | original | 20 | 1 | 95.0 |
|  | A | 20 | 2 | 90.0 |
|  | B | 20 | 1 | 95.0 |
|  | C | 20 | 3 | 85.0 |
|  | D | 20 | 2 | 90.0 |
| Hwanggeumal | original | 20 | 3 | 85.0 |
|  | A | 20 | 0 | 100.0 |
|  | B | 20 | 3 | 85.0 |
|  | C | 20 | 2 | 90.0 |
|  | D | 20 | 1 | 95.0 |
| Average | original | 20 | 1 | 93.8 |
|  | A | 20 | 2 | 92.5 |
|  | B | 20 | 1 | 93.8 |
|  | C | 20 | 2 | 91.3 |
|  | D | 20 | 2 | 92.5 |
